# Supplementary material for: Wuschel2 enables highly efficient CRISPR/Cas-targeted genome editing during rapid de novo shoot regeneration in sorghum
Source: Commun Biol. 2022 Apr 11;5:344. doi: 10.1038/s42003-022-03308-w (PMC9001672; doi:10.1038/s42003-022-03308-w)
Supplement: Supplementary file 2 — SUPPLEMENTAL MATERIAL [file 42003_2022_3308_MOESM2_ESM.pdf]

Supplementary information

a pPHP79066

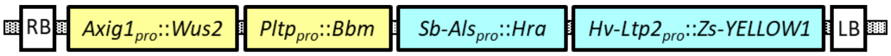

b pPHP8181

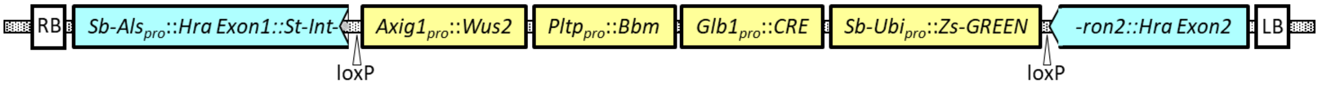

c pPHP94632

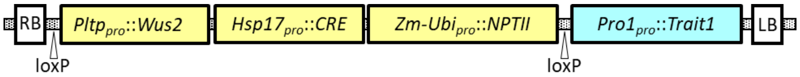

d pPHP94292

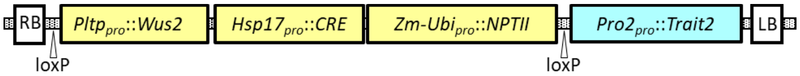

e PHP87078 (“Altruistic”)

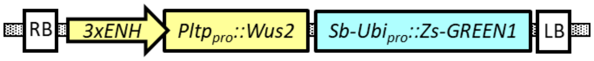

f pPHP88158 (“Altruistic”)

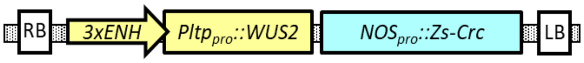

g pPHP96564

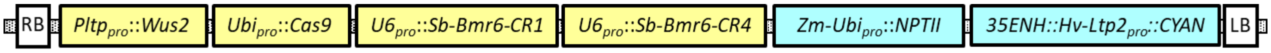

h pPHP86655

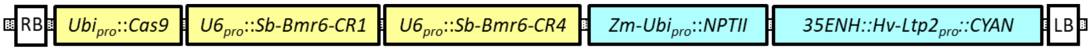

i pPHP87980

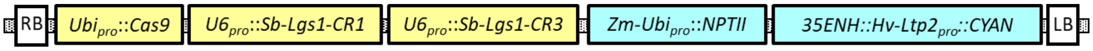

j pPHP86801

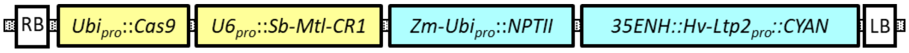

k pPHP87098

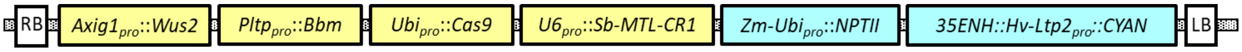

l pPHP87018

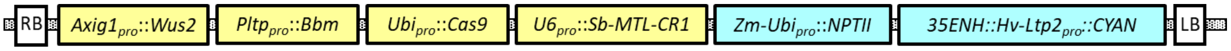

m pPHP87984

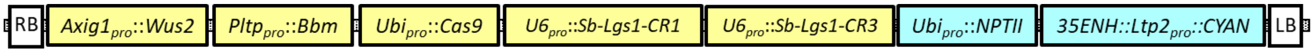

Supplementary Figure 1 Schematic representation of the molecular components of constructs used in this study.

Transformation procedures

Infection on day 1: Inoculate immature embryos with *Agrobacterium* LBA4404 Thy- harboring ternary vector system in infection medium (**Supplementary Table 1**)

Co-cultivation on day 1: Cultivate immature embryos with *Agrobacterium* on co-cultivation medium (**Supplementary Table 2**) at 25°C overnight followed by 28°C for 6 days in darkness

Conventional

Callus proliferation w/o selection on day 8: Induce embryogenic callus on the multi-purpose medium (**Supplementary Table 3**) at 28°C in darkness for a week

Callus proliferation w/ selection on day 15: Continue callus development on callus induction medium (**Supplementary Table 4**) with proper selection reagent at 28°C in darkness for total 9 weeks with subculturing every 3 weeks

Morphogenic gene-enabled

Somatic embryo formation w/o selection on day 8: Induce somatic embryo formation on the multi-purpose medium (**Supplementary Table 3**) at 28°C in darkness for a week

***Wus2/CRE*-enabled marker-free**

Somatic embryo formation w/ selection on day 8: Induce somatic embryo formation on the multi-purpose medium (**Supplementary Table 3**) with 250 mg/l G418 at 28°C in darkness for 3 weeks

Heat-shock on day 29: Incubate at 45°C for **3h**

Maturation w/o selection on day 29: Shoot regeneration on selection-free maturation medium (**Supplementary Table 5**) at 28°C in darkness for 4 weeks

Maturation w/ selection on day 78 or day 15: Shoot regeneration on maturation medium (**Supplementary Table 5**) with proper selection reagent at 28°C in darkness for 4 weeks

Rooting on day 106 or day 43 or day 57: Root regeneration on selection-free rooting medium (**Supplementary Table 6**) under light for 2 weeks

Rooted plants transferred to greenhouse on day 120 or day 57 or day 71

Supplementary Figure 2 Flow diagram of the *Agrobacterium*-mediated transformation systems.

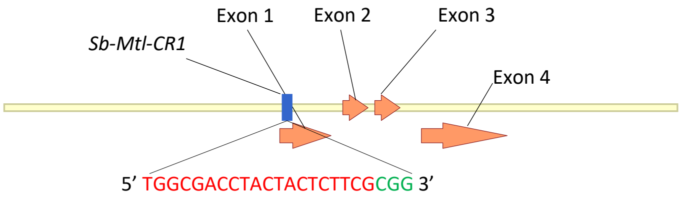

Supplementary Figure 3 Diagram of the *Sb-Mtl* gene structure and target site. Gene structure of *Sb-Mtl* with sgRNA target site (in red) and PAM sequence (in green).

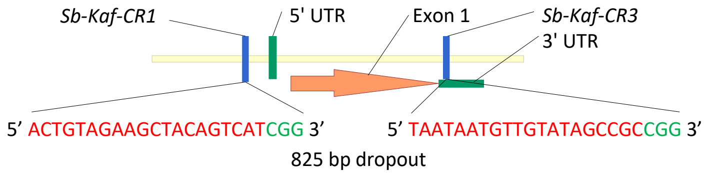

Supplementary Figure 4| Diagram of the *Sb-Kaf* gene structure and target sites. Gene structure of *Sb-Kaf* with sgRNA target sites (in red) and PAM sequences (in green).

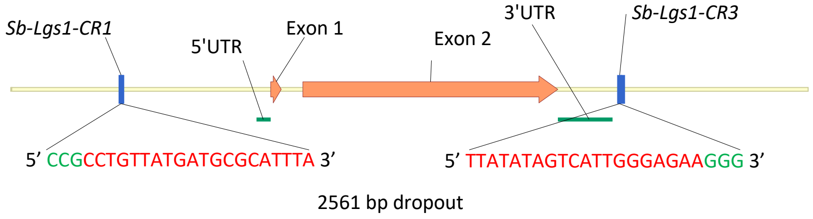

Supplementary Figure 5 Diagram of the *Sb-Lgs1* gene structure and target sites. Gene structure of *Sb-Lgs1* with sgRNA target sites (in red) and PAM sequences (in green).

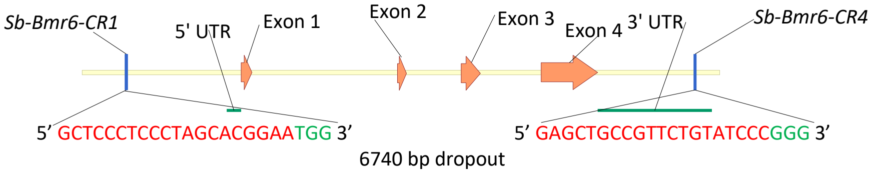

Supplementary Figure 6 Diagram of the *Sb-Bmr6* gene structure and target sites. Gene structure of *Sb-Bmr6* with sgRNA target sites (in red) and PAM sequences (in green).

Supplementary Table 1 Infection medium

MS salt	4.3 g/l
Myo-Inostitol	0.1 g/l
Nicotinic acid	0.5 mg/l
Pyridoxine HCl	0.5 mg/l
Thiamine HCl	1 mg/l
Casamino acids	1 g/l
Dichlorophenoxyacetic acid (2,4-D)	1.5 mg/l
Sucrose	68.5 g/l
Glucose	36 g/l
Thymidine	50 mg/l
Acetosyringone	100 μM (Add fresh)
Silwet L-77	0.005%
Adjusted OD for *Agrobacterium*	1.0

Supplementary Table 2 Co-cultivation medium

MS salt	4.3 g/l
Myo-Inostitol	0.1 g/l
Nicotinic acid	0.5 mg/l
Pyridoxine HCl	0.5 mg/l
Thiamine HCl	1 mg/l
Dichlorophenoxyacetic acid (2,4-D)	2 mg/l
Sucrose	20 g/l
Glucose	10 g/l
L-Proline	0.7 g/l
MES Buffer	0.5 g/l
Sigma Agar	8 g/l
Acetosyringone	100 μM (Add fresh)
Ascorbic acid	10 mg/l
Thymidine	50 mg/l

Supplementary Table 3 Multi-purpose medium

MS salt	4.3 g/l
Myo-Inostitol	0.25 g/l
Casein hydrolysate	1 g/l
Thiamine HCl	1 mg/l
Dichlorophenoxyacetic acid (2,4-D)	2 mg/l
Maltose	30 g/l
L-Proline	0.69 g/l
Cupric sulfate	1.22 mg/l
Phytagel	3.5 g/l
BAP	0.5 mg/l
Carbenicillin	100 mg/l
Selection reagent*	

*Either no selection or 250mg/l G418 or 0.1mg/l imazapyr.

Supplementary Table 4 Callus proliferation medium

MS salt	4.3 g/l
Myo-Inostitol	0.25 g/l
Casein hydrolysate	1 g/l
Thiamine HCl	1 mg/l
Dichlorophenoxyacetic acid (2,4-D)	1 mg/l
Maltose	30 g/l
L-Proline	0.69 g/l
Cupric sulfate	1.22 mg/l
Phytagel	3.5 g/l
BAP	0.5 mg/l
Carbenicillin	100 mg/l
Selection reagent*	

* Either no selection or 250mg/l G418 or 0.1mg/l imazapyr.

Supplementary Table 5 Maturation medium

MS salt	4.3 g/l
Myo-Inostitol	0.1g/l
MS vitamins stock (0.1 g/l nicotinic acid, 0.1 g/l pyridoxine HCl, 0.02 g/l thiamine HCl, 0.4 g/l glycine)	5 ml/l
zeatin	0.5 mg/l
Cupric sulfate	1.25 mg/l
L-Proline	0.7 g/l
Sucrose	60 g/l
Phytagel	3.5 g/l
Indole-3-acetic acid	1 mg/l
ABA	0.1 µM
Thidiazuron	0.1 mg/l
Carbenicillin	100 mg/l
Selection reagent*	

* Either no selection or 250mg/l G418 or 0.1mg/l imazapyr.

Supplementary Table 6 Rooting medium

MS salt	2.15 g/l
Myo-Inostitol	0.05 g/l
MS vitamins stock (0.1 g/l nicotinic acid, 0.1 g/l pyridoxine HCl, 0.02 g/l thiamine HCl, 0.4 g/l glycine)	2.5 ml
Sucrose	20 g/l
Phytagel	3 g/l

Supplementary Table 7 The integrated morphogenic gene-enabled transformation efficiencies of six Corteva elite lines

Genotype	# of embryos infected	# of T0 plants	Transformation efficiency (%)
PH1679	280	4	1.4
PH1719	233	47	20.1
PH1181	240	16	6.7
PH2517	162	29	17.9
PH1481	235	54	23
PH2545	226	56	24.7

Supplementary Table 8 *Sb-Mtl* mutation frequency using single gRNA in Tx430

Transformation system	Construct	sgRNA	# T0 plants analyzed	# of T0 events w/ mutation (freq.)
Conventional	pPHP86801	*Sb-Mtl-CR1*	29	8 (27.5%)
Integrated morphogenic gene-enabled	pPHP87098 (*Axig1*$_{pro}$*:Wus2/Pltp*$_{pro}$*:Bbm*)	*Sb-Mtl-CR1*	97	72 (74.2%)

Supplementary Table 9 *Sb-Kaf* mutation and gene-dropout frequencies using two gRNAs in Tx430

Transformation system	Construct	sgRNA	# T0 plants analyzed	# of T0 events w/ mutation (freq.)	# T0 events w/ gene-dropout (freq.)
Conventional	pPHP87018	*Sb-Kaf-CR1*	33	13 (39.3%)	0
		Sb-Kaf-CR3		16 (48.5%)	
Non-integrated *Wus2*-enabled altruistic	(90%) pPHP87018+(10%) PHP88158 (*3xENH:Pltp*$_{pro}$*:Wus2/NOS*$_{pro}$*:Crc*)	*Sb-Kaf-CR1*	33	21 (63.6%)	1 (3.0%)
		Sb-Kaf-CR3		22 (66.7%)	

Supplementary Table 10 *Sb-Lgs1* mutation and gene-dropout frequencies using two gRNAs in Macia

Transformation system	Construct	sgRNA	# T0 plants analyzed	# of T0 events w/ mutation (freq.)	# T0 events w/ gene-dropout (freq.)
Integrated morphogenic gene-enabled	PHP87984 (*Axig1*$_{pro}$*:Wus2/Pltp*$_{pro}$*:Bbm*)	*Sb-Lgs1-CR1*	42	35 (83.3%)	22 (52.4%)
		Sb-Lgs1-CR3		39 (92.9%)	
Non-integrated *Wus2*-enabled altruistic	(90%) PHP87980+(10%) PHP88158 (*3xENH:Pltp*$_{pro}$*:Wus2/NOS*$_{pro}$*:Crc*)	*Sb-Lgs1-CR1*	161	65 (40.4%)	47 (29.2%)
		Sb-Lgs1-CR3		93 (57.8%)	
